# Phyllodes Tumor of the Breast Complicated With Mastitis

**DOI:** 10.7759/cureus.45206

**Published:** 2023-09-14

**Authors:** Siddharth Sankar Das, Akshata Mestha, Sahil Navlani, Esaaf Hasan Ghazi Mohd

**Affiliations:** 1 General Surgery, Dubai Hospital, Dubai, ARE; 2 General Practice, Dubai Academic Health Corporation, Dubai, ARE

**Keywords:** incision and drainage, postpartum, pregnancy, mastitis, breast mass, benign neoplasm, fibro-epithelial lesion, phyllodes tumor

## Abstract

One of the rarest fibro-epithelial neoplasms of the breast during pregnancy is the phyllodes tumor (PT). It is typically a painless, bi-phasic, and rapidly growing neoplasm that resembles fibroadenomas. It is still unclear if the neoplasm is hormone-dependent during pregnancy. It is often challenging to diagnose and treat PT. Herein, we report a case of a 30-year-old female at 31 weeks gestation who was diagnosed with a benign phyllodes tumor of her breast with concurrent mastitis. She was first seen during her third trimester where the neoplasm was around 5 cm as reported by the ultrasound (US) examination. Her biopsy report was suggestive of a PT and she was advised surgery with excision of the tumor margin, but she refused. Ten days after her delivery she presented to the emergency department with a fever and a hard, engorged, erythematous, and tender left breast. She was diagnosed with mastitis of the left breast. She then underwent incision and drainage of the left breast that drained purulent milk; additionally, large necrotic grape-like tissues were removed and were confirmed by the histopathology report as a benign phyllodes tumor of the breast.

## Introduction

The breast is located in the pectoral region superficial to the pectoral fascia and pectoral group of muscle. PT is an uncommon tumor of the breast that constitutes less than 1% of all breast neoplasms [1-7] and around 2.5% of all the fibro-epithelial lesions of the breast [8-10]. The neoplasm was first reported in 1774, but it was named in 1838 as cystosarcoma phyllodes by Johannes Muller and later renamed in 1982 by the World Health Organization as phyllodes tumor [1,11]. It is usually seen in females of the reproductive age group [2-4]. The development of PT during pregnancy is very unusual and it can quickly grow into a larger neoplasm [7-10,12,13]. Approximately 20% of the neoplasms are diagnosed by screening mammograms as lobulated masses [11]. PTs that are greater than 10 cm are considered giant in size and constitute around 20% of all the phyllodes neoplasms [5,10]. However, it has also been reported to grow as big as 60 cm [12].

## Case presentation

A 30-year-old primigravida female at 31 weeks gestation presented with complaints of painful left breast mass and enlargement since her second trimester. She has no previous history of breast surgeries, no breast lumps in the past, and no positive family history. On examination, the left breast was engorged and a soft, firm, mobile, non-tender lobulated mass was palpable at the inferior-lateral region. The right breast was normal. 

**Figure 1:**
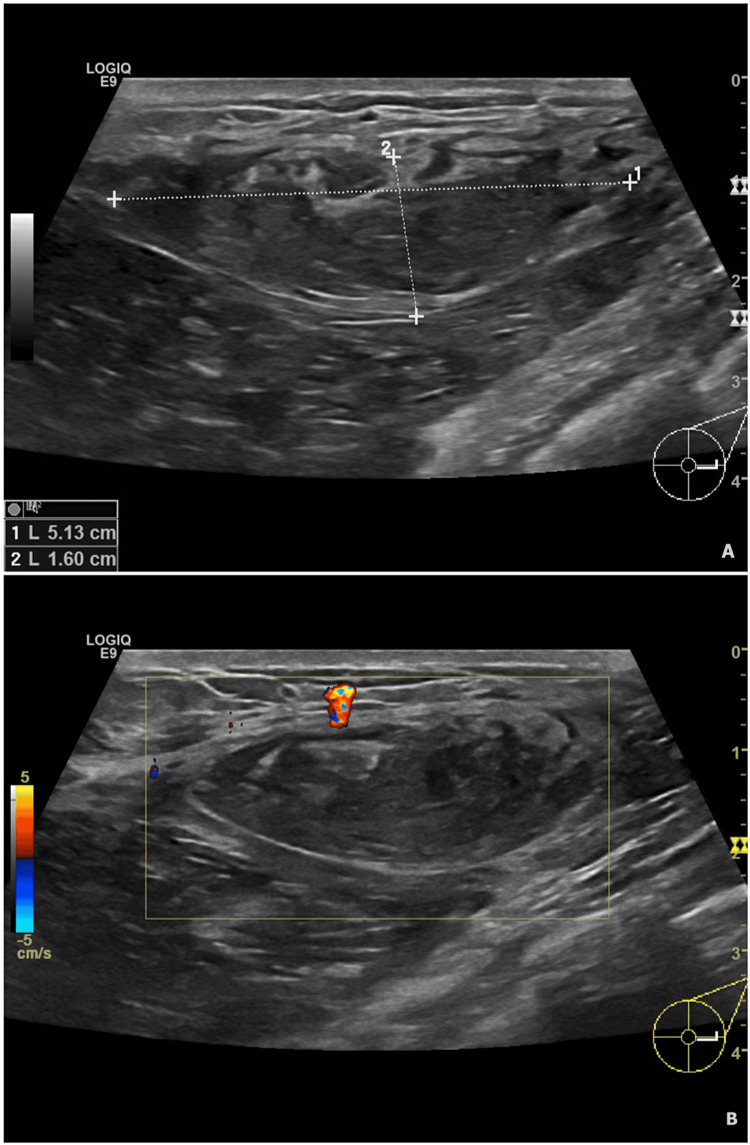
US examination of the left breast (A) In the heterogeneous fibro-glandular left breast, a 5.13 cm x 1.6 cm circumscribed parallel hypoechoic lesion extends from three to nine o’clock, periareolar to peripherally, showing heterogeneous echotexture. (B) Vascular lesion of the left breast.

A US examination of the breast was done which demonstrated a large lesion occupying the lower half of the left breast (Figure 1) displacing the nipple upward with no ductal dilatation. The Breast Imaging Reporting and Data System (BI-RADS) score was four. No pathological axillary lymph nodes were detected.

**Figure 2:**
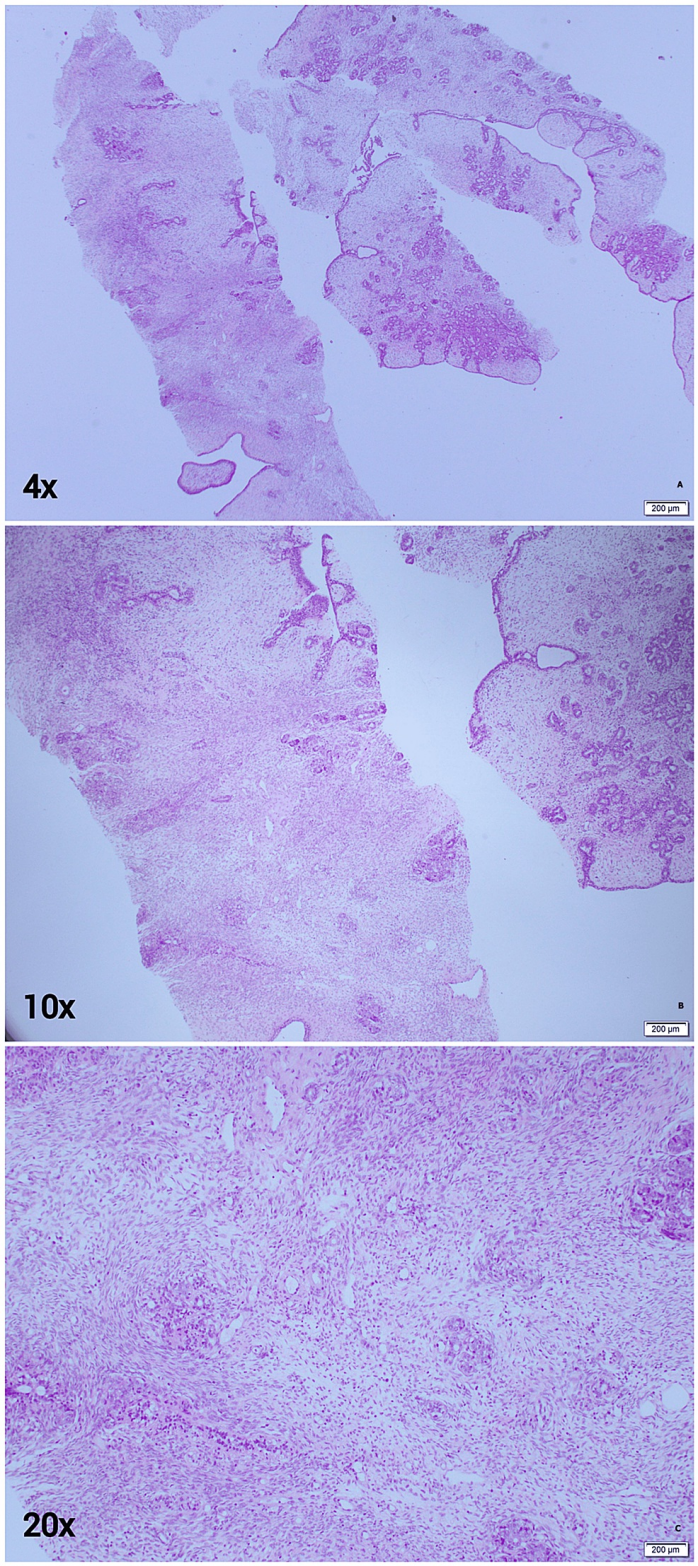
Breast tissue sections at 4x, 10x and 20x magnifications, showing a cellular fibroepithelial lesion. The epithelial-to-stromal ratio is disrupted due to a marked increase in stromal cellularity. There is stromal condensation around the peri-ducts with a scattering of lymphocytes in the stroma and absence of mitosis, cytological atypia, and necrosis.

She was then advised to undergo a US examination-guided biopsy. The biopsy report showed a cellular fibroepithelial lesion of the left breast (Figure 2), suggesting a phyllodes tumor or a cellular fibroadenoma (FA). The decision for surgery by wide local excision with negative margins was taken at the multidisciplinary team (MDT) meeting. The patient was counseled to undergo surgery before her delivery but she refused.

**Figure 3:**
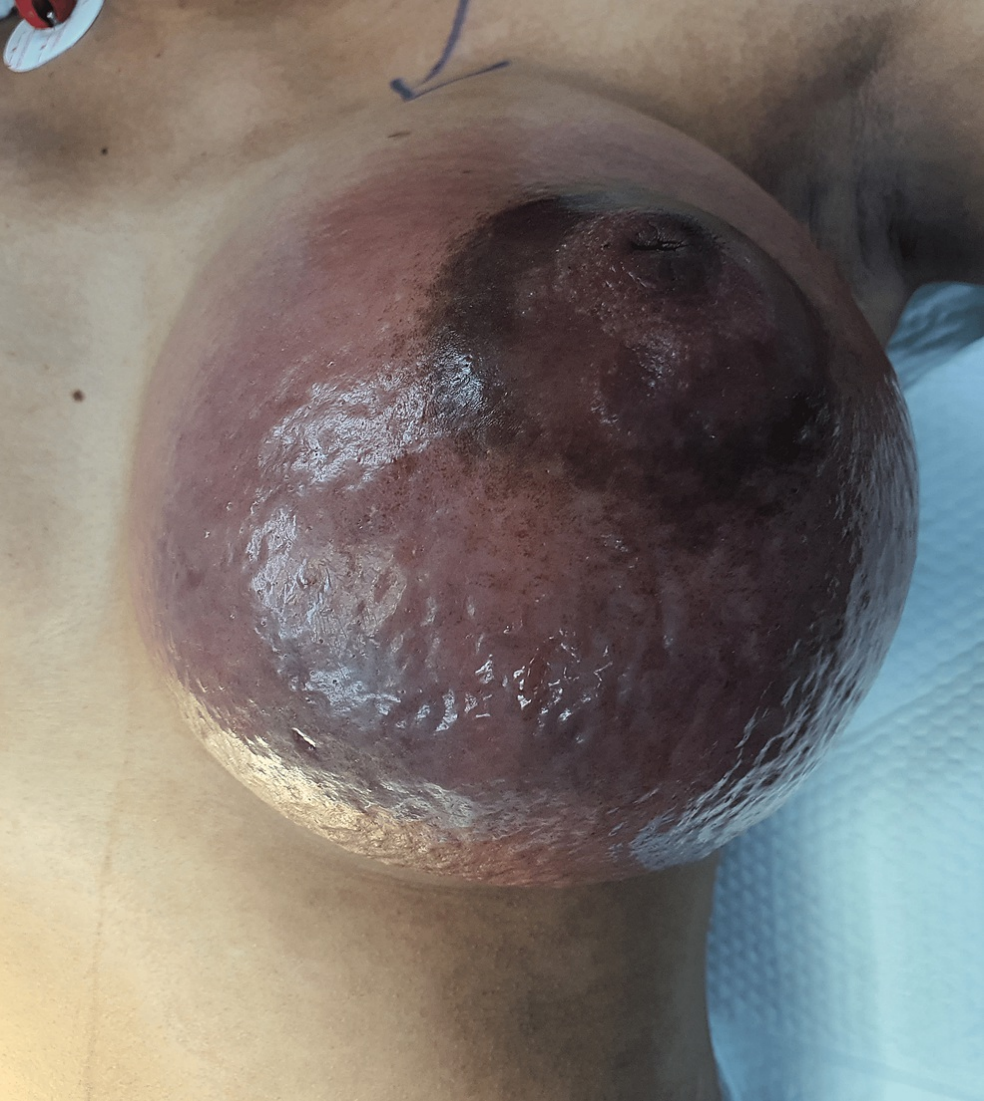
Engorged and erythematous left breast.

**Figure 4:**
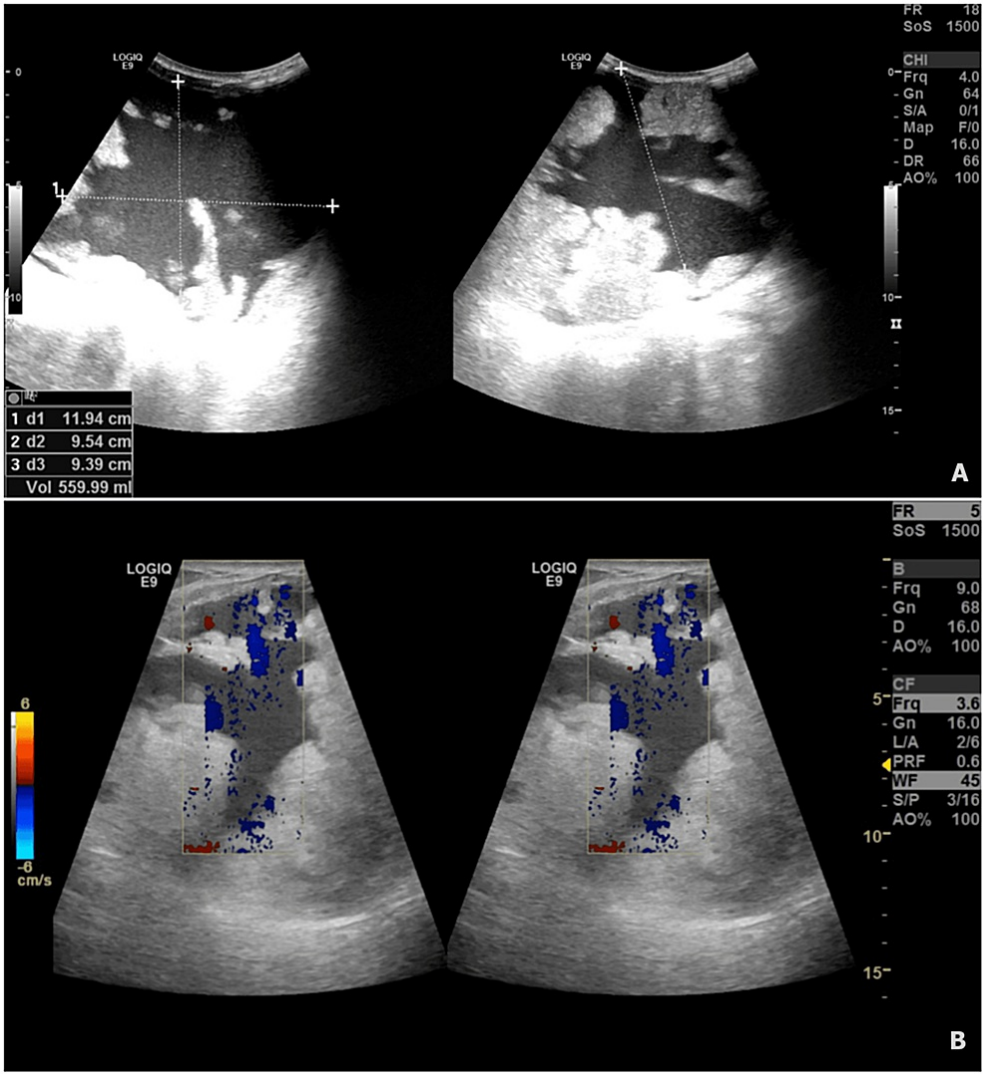
US examination of the left breast (post-delivery). (A) Left breast collection measuring 11.94 cm x 9.54 cm x 9.39 cm. (B) Highly vascular collection of the left breast.

Ten days post-delivery she presented to the emergency department with breast engorgement and pain for four days and fever for one day. There was no milk expressed from the left breast except for a few drops. She had done a US examination in a private hospital that showed a solid cystic lesion measuring around 17.5 cm x 13.7 cm x 12.8 cm occupying all quadrants of the left breast. On examination the right breast was normal and the left breast was hard, engorged, erythematous, and tender with mild fluctuation that was felt at the lower aspect. There were no nipple changes or discharges (Figure 3). Her vital signs showed a fever of 38.4 °C. She then underwent an urgent US examination of the breast that showed a large irregular collection with low-level internal echoes in the left breast suggestive of an abscess, most likely mastitis (Figure 4). The collection had multiple extensions within the glandular tissue.

**Figure 5:**
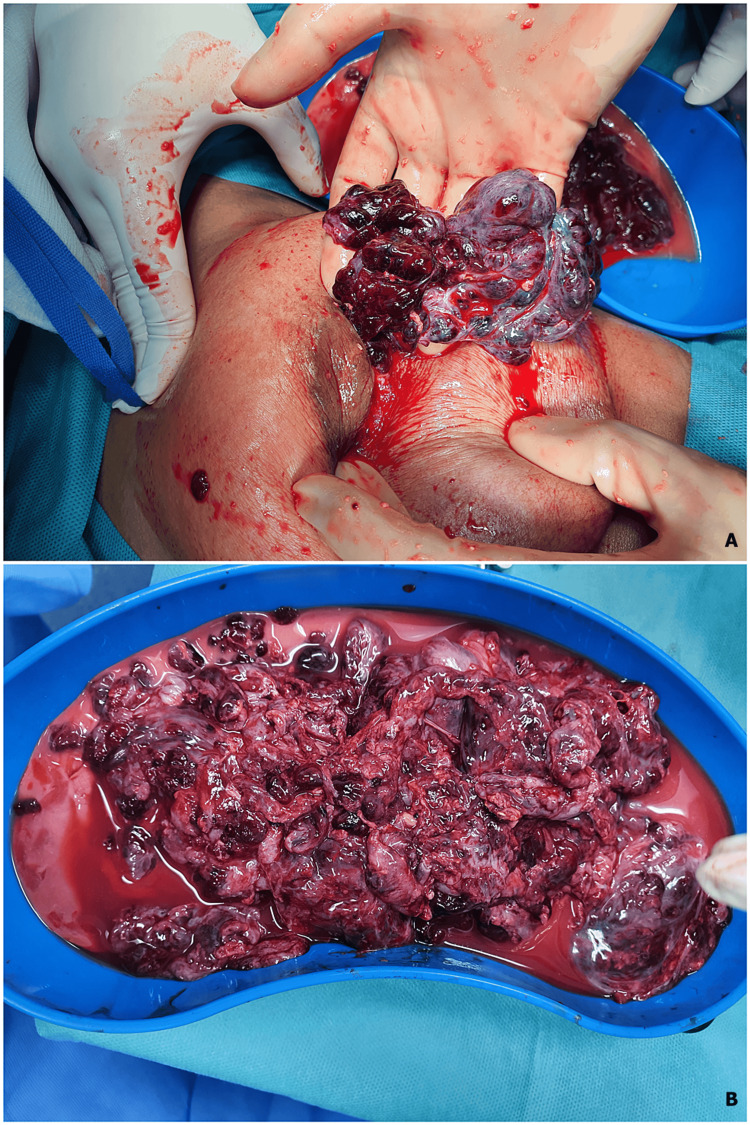
Phyllodes tumor visualized as grape-like tissues. (A) Finger swapping of the tumor from the left breast. (B) Phyllodes tumor that is visualized as grape-like tissues.

Later that evening, she was taken to the operation theater for incision and drainage of the collection. A circumocular incision was made longitudinally along the three to five o'clock position that drained around 600 ml of purulent milk. All locules were broken using a finger. Large necrotic grape-like tissues were removed by finger swapping (Figure 5). The cavity was irrigated with Eusol, Dermacyn then Betadine solution and packed with Betadine-soaked dressing. There was around 50-70 ml of blood loss mixed with milk.

Postoperatively the patient was doing well and breastfed through the right breast. The wound was regularly cleaned with normal saline and betadine then packed with Aquacel dressing until vacuum-assisted closure (VAC) was applied three days later. She was discharged six days after the surgery with a follow-up in the breast clinic.

**Figure 6:**
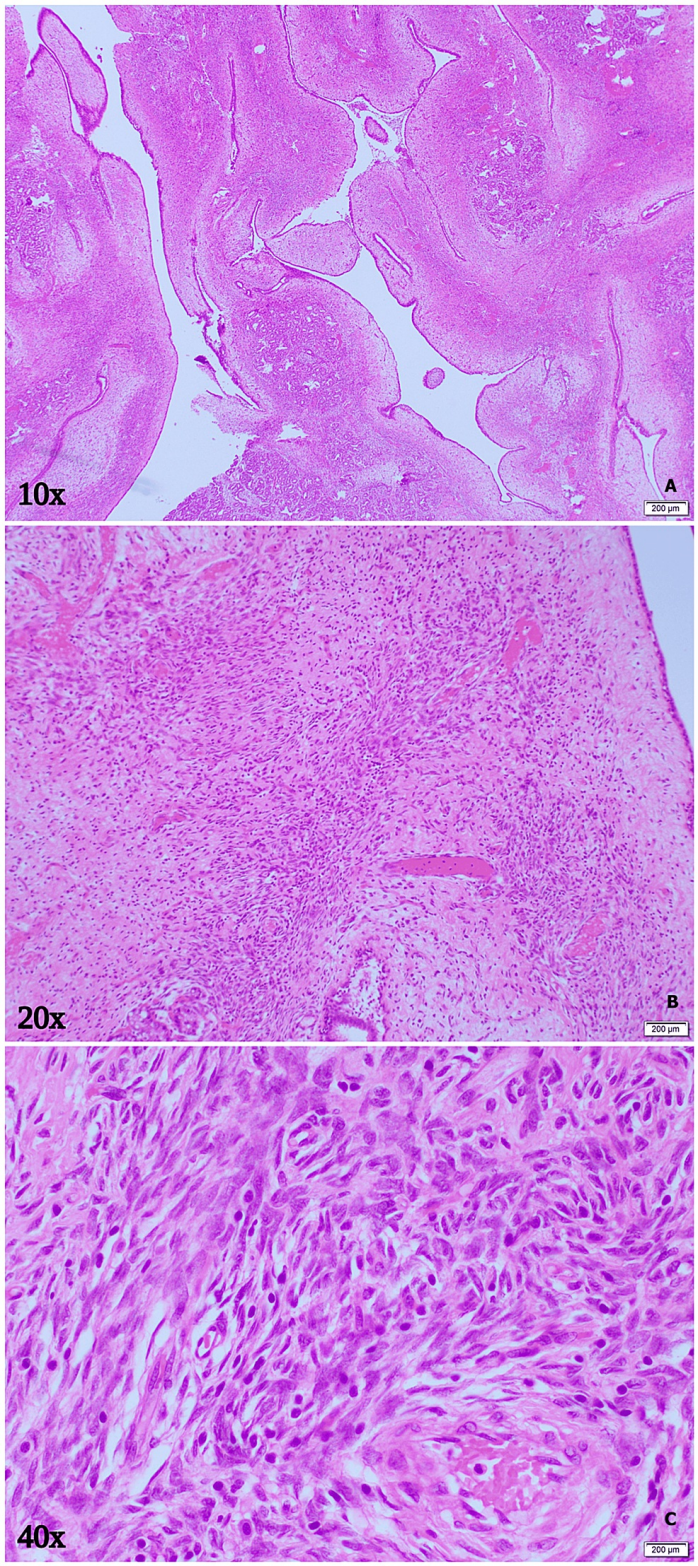
Breast tissue sections at 10x, 20x, and 40x magnifications, showing leaf-like ductal spaces lined by bilayered epithelial and myoepithelial cells. There are foci showing periductal stromal accentuation. There is mildly increased stromal cellularity and mild nuclear atypia. Mitoses are rare, around 1-2 per 10 high-power fields. Adjacent breast parenchyma showed secretory changes in the lobules and acini with scant intervening stroma. There are extensive areas of hemorrhage with granulation tissue formation. No brisk mitotic activity, marked stromal overgrowth and atypia, infiltrative edge, or necrosis is seen.

On follow-up, the wound culture did not show any organisms. The histopathology report showed a cellular fibroepithelial neoplasm with features of benign phyllodes tumor of the breast (Figure 6). The decision to schedule close follow-ups was made at the MDT meeting. At the fourth follow-up, the patient had an induration at the lower quadrant of the left breast with a mass and the surgical wound was small with dimpling of the skin. To rule out residual or recurrence of the phyllodes tumor she was advised another US examination and biopsy but the patient decided to continue her follow-ups in her home country.

## Discussion

PT is one of the rarest fibro-epithelial neoplasms of the breast during pregnancy [4,6,7,10,13]. The onset of PT varies in different patients, and it could be from one week to six years. It commonly presents in the postpartum period, followed by the second trimester of pregnancy [5]. 

It is still unknown if the neoplasm is hormone-dependent in pregnancy [4-6,9,10,14]. Various researchers have examined the presence of estrogen and progesterone receptors in the neoplasm, but no conclusive result exists [8,9]. Researchers have speculated that they promote the growth of PT [5,6,8,14]. 

During pregnancy, hormonal changes cause the breasts to undergo physiological alterations, such as the proliferation of lobules and alveoli [2,6,13] and vascular hyperplasia, leading to increased breast weight, volume, density, and firmness [5,7,14]. Due to hormonal changes during gestation, it becomes challenging to detect breast masses in women [4,5,7,11,14].

PT was generally self-reported in the past, but nowadays, it is detected by imaging studies [5]. Clinical breast examinations are recommended early in pregnancy for early diagnosis of the neoplasm [7]. As per Alipour et al., the neoplasm presented as a mass in 90.7% of the patients and as a breast enlargement in 9.3% [5]. It is a solitary, firm, rapidly growing, painless, mobile neoplasm with well-defined edges [3,4,12,14]. It is usually covered by a false capsule [14] and grows radially, extending into the healthy tissues and compressing the adjacent breast tissue [2]. It can also present with dilated veins on the skin [2], skin discoloration, inflammatory changes [3,4], and skin ulcers [5]. A study by Adjoby et al. reported unilateral neoplasms as large as 20 cm in diameter [4], whereas another study found it was about 45 cm in diameter [3]. Unilateral PT can occur in the right or left breast without preference [5]. Bilateral PT has been seen in about 16% of the cases [4,5]. On mammography, it is described as a lobulated, well-circumscribed opacity [7]. Ultrasound of the neoplasm shows heterogeneous echoic structures mixed with cystic anechoic areas [1,3,4,7]. The gold standard for diagnosis is by histopathology [4,12,14].

Histologically, PT has been categorized into three groups, namely benign, borderline, or malignant, based on the extent of the cellularity of the stroma, presence or absence of atypical cells, proliferation of the stroma, mitotic activity and the nature of the margin of the neoplasm [2-4,8,12-14]. The histological differences between the three categories of PT have been described in Table 1 [12-14]. 

**Table 1:** Histological differences between the three categories of PT PT: phyllodes tumor; HPF: high power fields

Phyllodes tumor histological classification	Stromal Atypia	Stromal Overgrowth	Stromal Cellularity	Mitotic Activity	Infiltrative Borders
Benign	Mild	Absent	Mild	< 5 per 10 HPF	Absent
Borderline	Moderate	Absent or Focal	Moderate	5 - 9 per 10 HPF	Absent
Malignant	Marked	Present	Marked	> 10 per 10 HPF	Present

Macroscopically, the neoplasm is lobulated with a red or gray meaty texture with a smooth margin [1] and a cut section showing necrosis, hemorrhage, and fibro-gelatinous areas [12]. Generally, large neoplasms are heterogeneous and may have lactational changes, necrosis, and hemorrhage, especially in the postpartum period [10]. Microscopically, it is a leaf-like structure [12,13]. Since PT is a fibro-epithelial tumor, it resembles fibroadenomas, making it a possible differential diagnosis [1-4,6-8,11]. Several studies suggest that fibroadenoma can progress in a stromal direction to develop into a PT [8,15]. Some other differentials to be considered are lactating adenoma, galactocele, periductal stromal tumor [13], lipoma, hamartoma, and sarcoma [12].

Treatment with surgical-wide local excision [13] with a minimum of 1 cm of a clear surgical margin [1,2,6,8-12,14] is recommended for grade 1 and 2 neoplasms, and simple mastectomy without lymph node dissection is recommended for grade 3 neoplasms or those that are bigger than 5 cm in diameter [3-5,14]. On the other hand, a study reports that a 1 cm margin might amount to over-treatment as there is no difference in recurrence rates between a 0.1 cm and a 1 cm margin [16]. Since wide excision surgery may result in reduced breast volume and deformity, it could be linked to psychological issues and a lower quality of life. Therefore, to achieve improved cosmetic outcomes, Pankratjevaite et al. suggested considering staged excision [12]. The patients should be followed up regularly after the surgery [5] with repeated breast exams and imaging [2].

Adjuvant radiotherapy is known to reduce the local recurrence rate [6]. It is recommended for grade 3 neoplasms or after three local recurrences or one recurrence after mastectomy [3,4]. Although adjuvant chemotherapy has little clinical benefit [6], it can be offered to patients with neoplasms larger than 5 cm or recurrent malignant neoplasms [2]. Turalba et al. demonstrated that doxorubicin and ifosfamide-based chemotherapy significantly treat women with metastatic PT [17]. Chemotherapy can be done as needed during the second trimester of pregnancy. In contrast, radiotherapy is typically given postpartum, leading to special cases that may necessitate preterm delivery to ensure timely completion of adjuvant treatment and reduce the risk of recurrence [11]. Most studies do not advocate using adjuvant therapy for adequately resected neoplasm [10], and patients can be followed up annually [12]. Hormonal therapy is not effective in PT [2]. Insufficient treatment may lead to tumor recurrence, growth, or even metastatic spread, especially in cases where the tumor exhibits malignant characteristics [12].

Around 60% of the PT cases are benign in nature [8,13,14]. Usually, PT has a low potential for metastasis, but it has an increased recurrence rate [9,13]. Alipour et al. suggest that female sex hormones could potentially trigger the malignant transformation in benign PT [5]. It is hypothesized that local recurrence occurs due to incomplete excision of the neoplasm [4,7,9,14]. Lu et al. reported recurrence rates as 8% for benign PT, 13% for borderline, and 18% for malignant [18]. The histological grade, type of surgery, and surgical margin excision status were identified as risk factors for recurrence [6,19]. However, the neoplasm's age and size were not associated with the risk of local recurrence [18]. One study reported the local recurrence ranging from 14-29%, especially within two years of management [9], and another study reported 15-40% [11]. Metastasis was observed in 10-20% of the cases [4,11] and 25-40% in another study [20]. Metastasis is usually through the hematogenous route [14]. The lungs are the most frequently affected site of metastasis [11], followed by the skeleton, heart, and liver [2]. The most important prognostic factor for developing distant metastases and survival rate is stromal overgrowth [14]. The five-year survival rate is 90% for benign PT and 80% for malignant PT [2]. The five-year disease-free survival was 95.7% for benign PT, 82.5% for borderline, and 72.6% for malignant [20].

## Conclusions

Phyllodes tumors of the breast are rare, but they can be a challenging diagnosis. They may present during pregnancy and, if associated with mastitis, make the diagnosis even more difficult. The treatment of phyllodes tumors of the breast is surgical excision. The prognosis is generally good, with a low risk of recurrence. It is still unknown if the neoplasm is hormone-dependent; hence further studies need to be conducted.
